# Supplementary material for: The current landscape and emerging challenges of benchmarking single-cell methods
Source: Brief Bioinform. 2025 Oct 4;26(5):bbaf380. doi: 10.1093/bib/bbaf380 (PMC12495992; doi:10.1093/bib/bbaf380)
Supplement: Supplementary_material_bbaf380 [file supplementary_material_bbaf380.docx]

# Supplementary Material

###

### Supplementary Figures


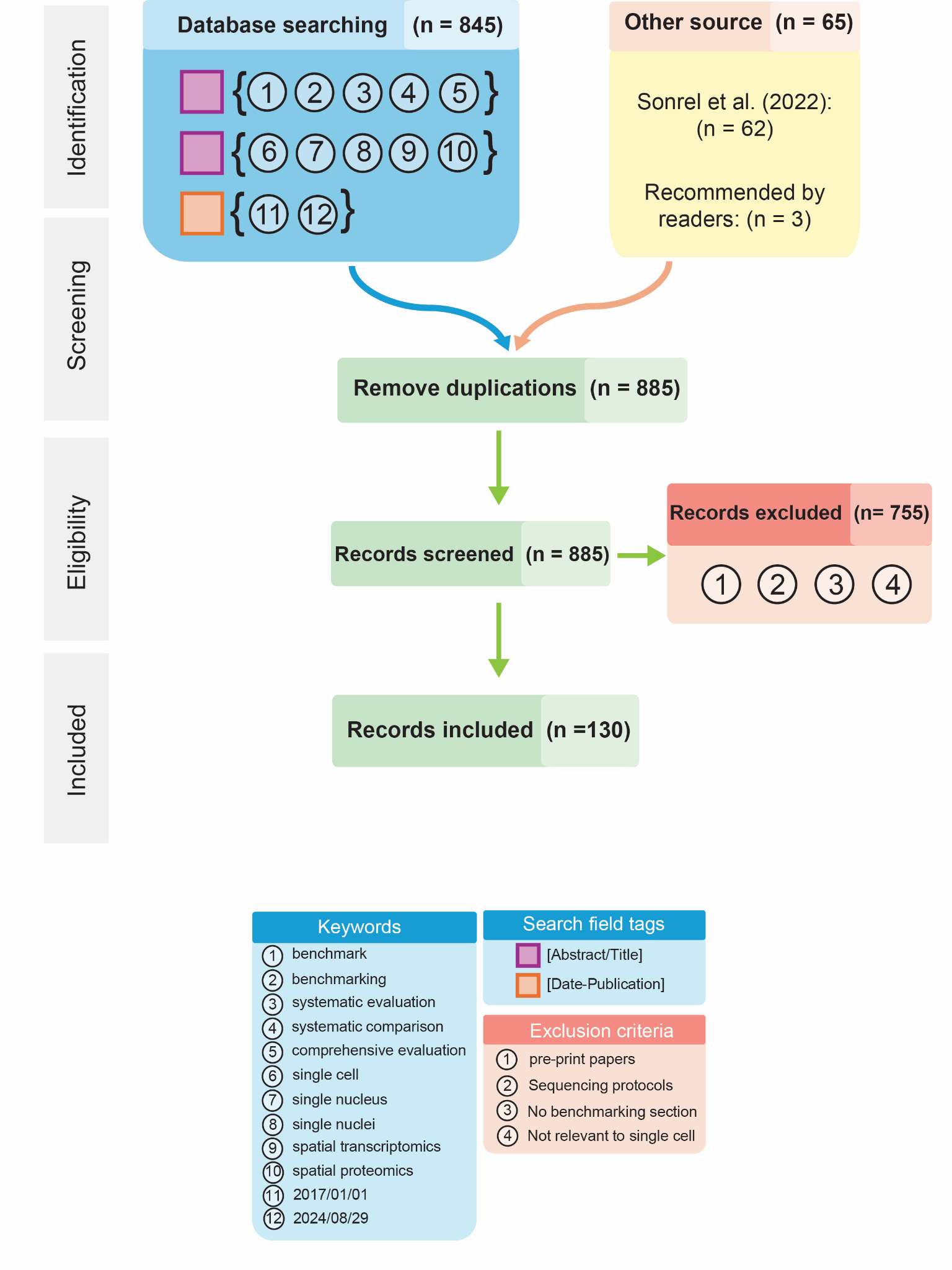


#### Supplementary Figure 1. Flowchart for systematic literature search of benchmark-only papers.

Using combinations of key terms for PubMed searches and multiple rounds of inclusion and exclusion procedures, we identified a total of 130 benchmark-only papers. For the exact search query used and details on each exclusion criterion, please see Methods section.


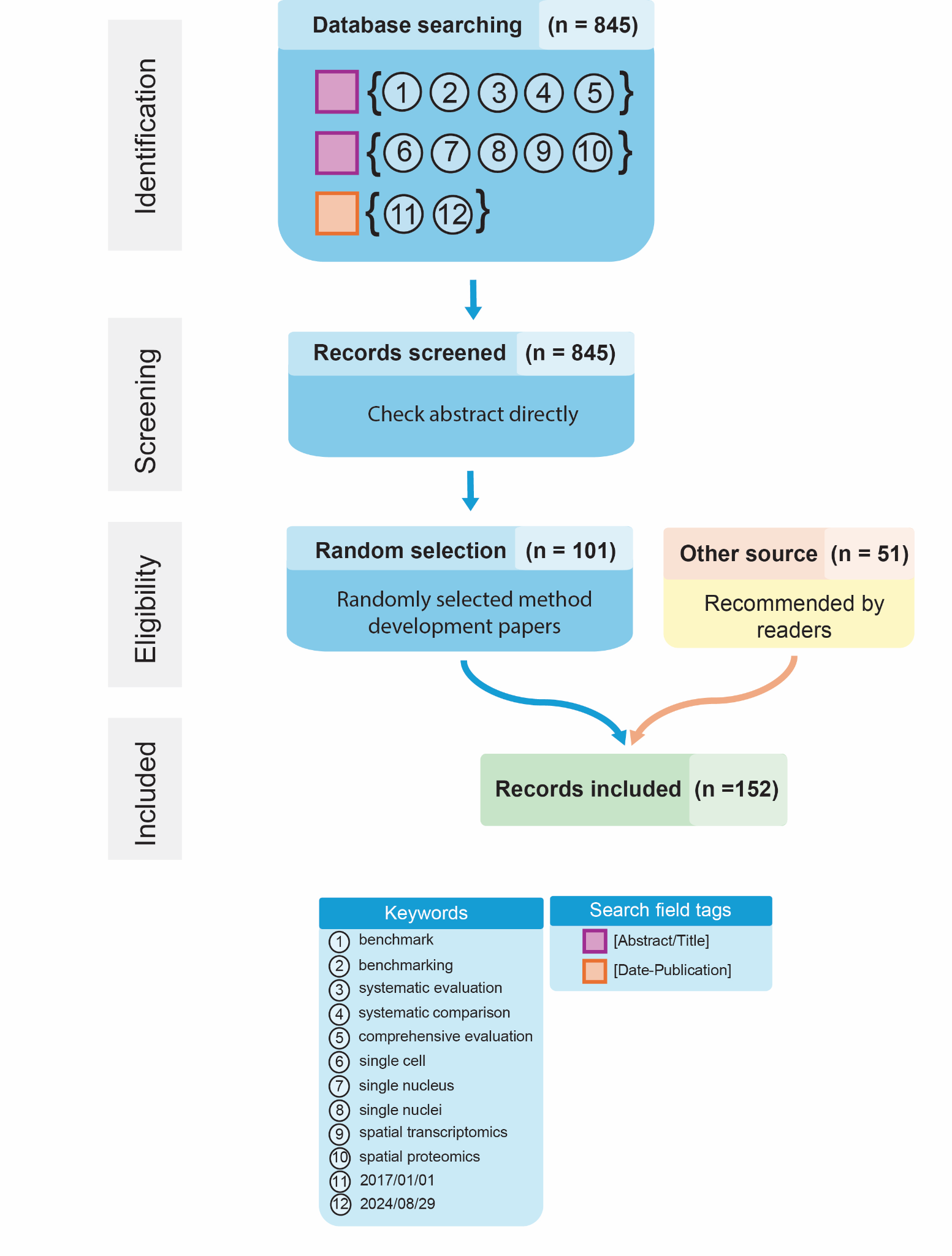


#### Supplementary Figure 2. Flowchart for systematic literature search of method development papers.

Using combinations of key terms for PubMed searches, random selection and reader recommendation, we identified a total of 152 method development papers. For the exact search query used and details on each exclusion criterion, please see Methods section.


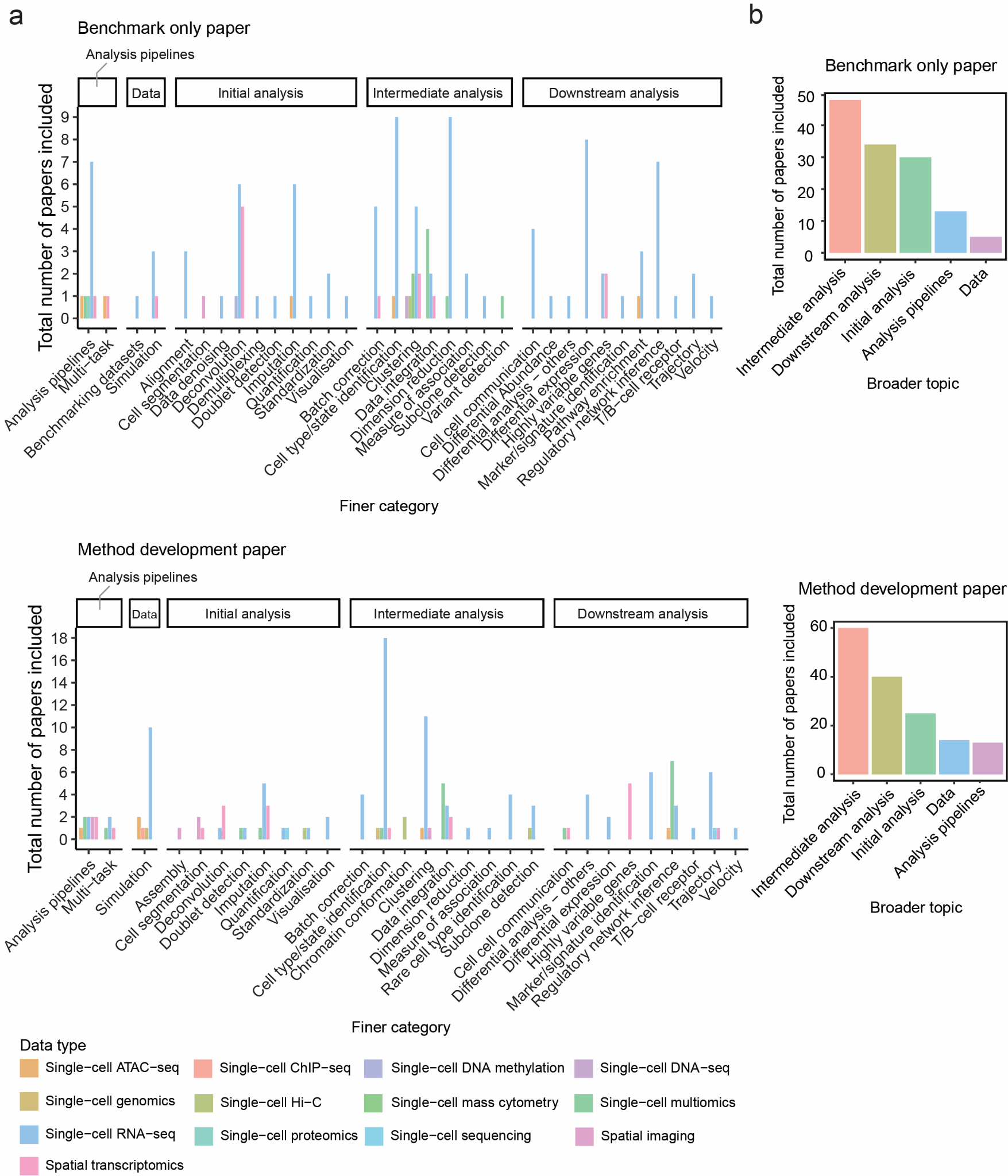


#### Supplementary Figure 3. Distribution of the papers across finer and broader categories.

a. Total number of benchmark-only and method development papers included in this study in each finer category, stratified by the broader category. Topics that are appear as top 10 frequent topics in both BOP and MDP includes analysis pipelines (BOP: n=11; MDP: n=9), clustering (BOP: n=11, MDP: n=13), cell type/state identification (BOP: n=10, MDP: n=21), data integration (BOP: n=7, MDP: n=10), imputation (BOP: n=7, MDP: n=9) and regulatory network inference (BOP: n=7, MDP: n=11). b. Total number of benchmark-only and method development papers in each broader category.


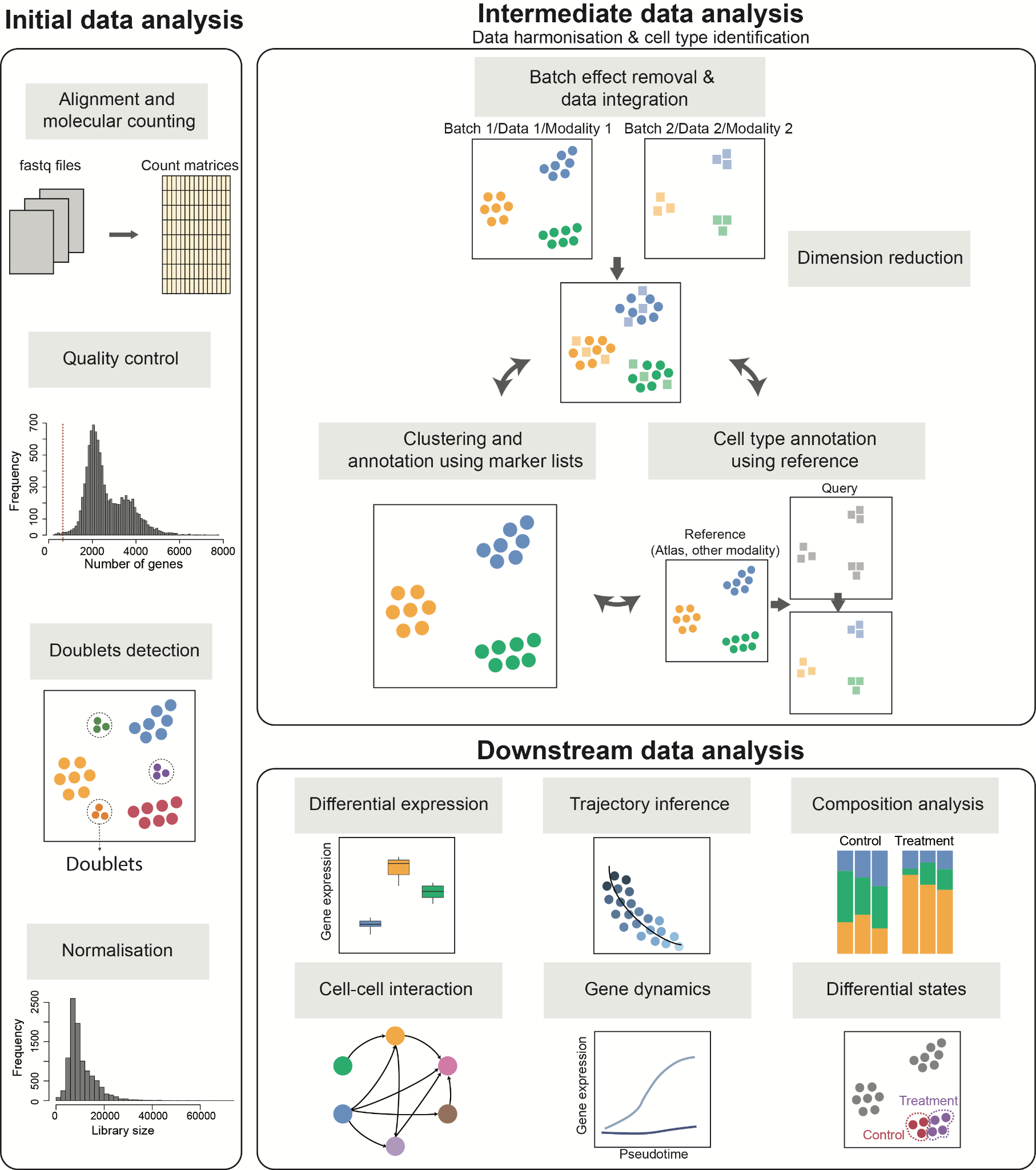


#### Supplementary Figure 4. Examples of initial data analysis, intermediate analysis and downstream data analysis.

In the study we classified the topic of each paper under five broad categories of “data”, “initial analysis”, “intermediate analysis” and “downstream analysis”. This figure gives selected examples for some of these categories. Initial data analysis includes data preprocessing such as alignment, quality control and doublet removal and ensures that only good quality cells are kept for analysis. Intermediate data analysis focuses on integrating data across modalities or batches when there is a batch effect and identification of cell populations through clustering or annotation approaches to facilitate downstream analysis. Downstream analysis includes various tasks that extract biological insights from the data to answer biological questions that motivated the experiment in the first place.


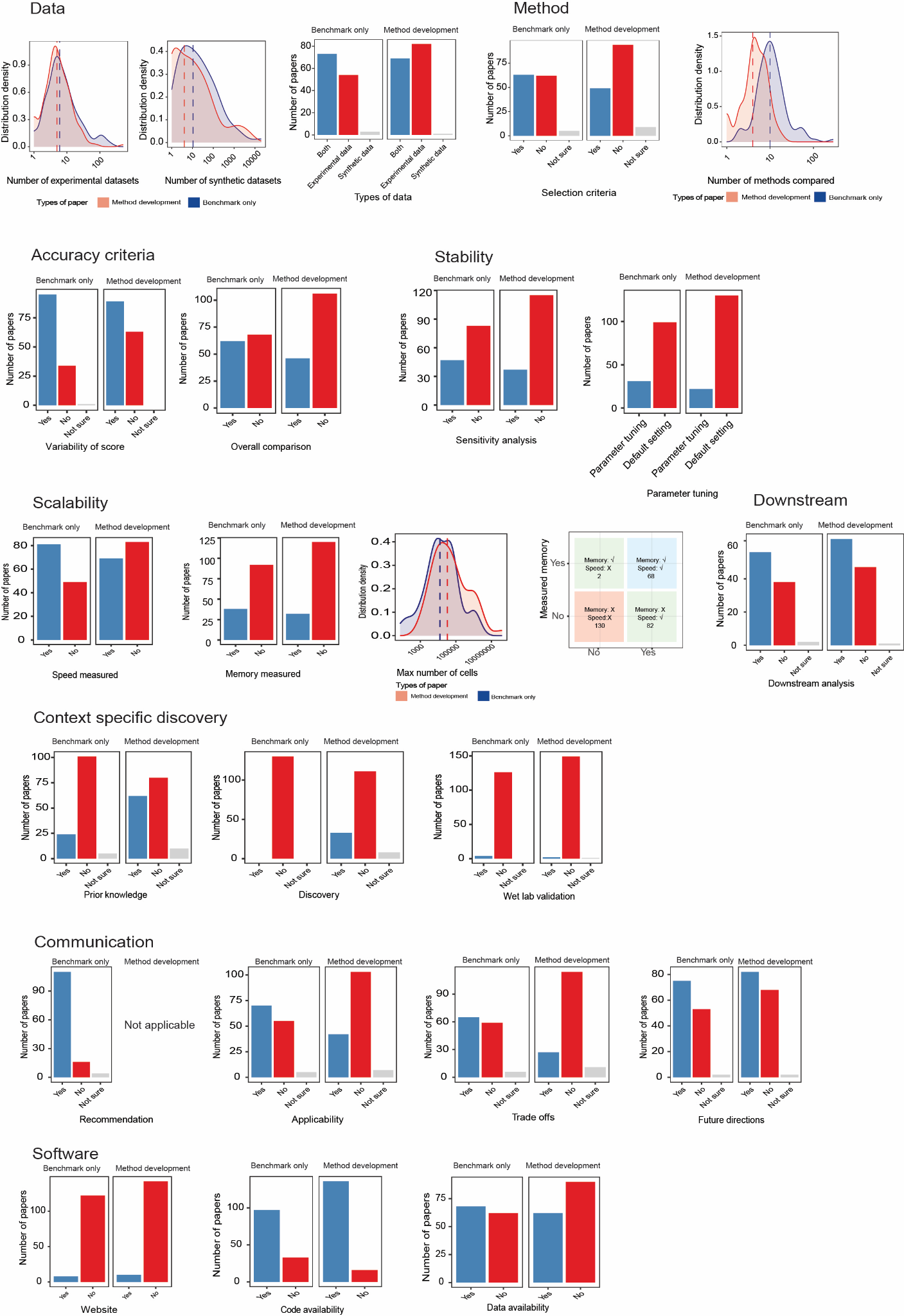


#### Supplementary Figure 5. Overview of survey response.

This figure provides a visual overview of the survey responses across the nine criteria categories. BOP and MDP use a similar number of experimental datasets, while BOP use more synthetic datasets. As expected, BOP compared with more methods on average, compared to MDP. MDP more often omit explicit rationale for the selection of methods compared to BOP. BOP more often report variability of scores and overall comparison across multiple performance metrics compared to MDP. Sensitivity analysis and varied parameter settings are more often omitted than included in both BOP and MDP, highlighting the need of more robust evaluation in future work. While on average more BOP compare the speed of methods than MDP, MDP tested on a greater number of cells. Memory is less frequently measured in both BOP and MDP. Both BOP and MDP include downstream task as part of their evaluation framework. Prior biological knowledge and discovery are more commonly discussed in MDP when interpreting the evaluation results, while applicability and trade-offs of methods are commonly discussed in BOP to aid user decision making. While code is often provided in both BOP and MDP, processed data is less available.


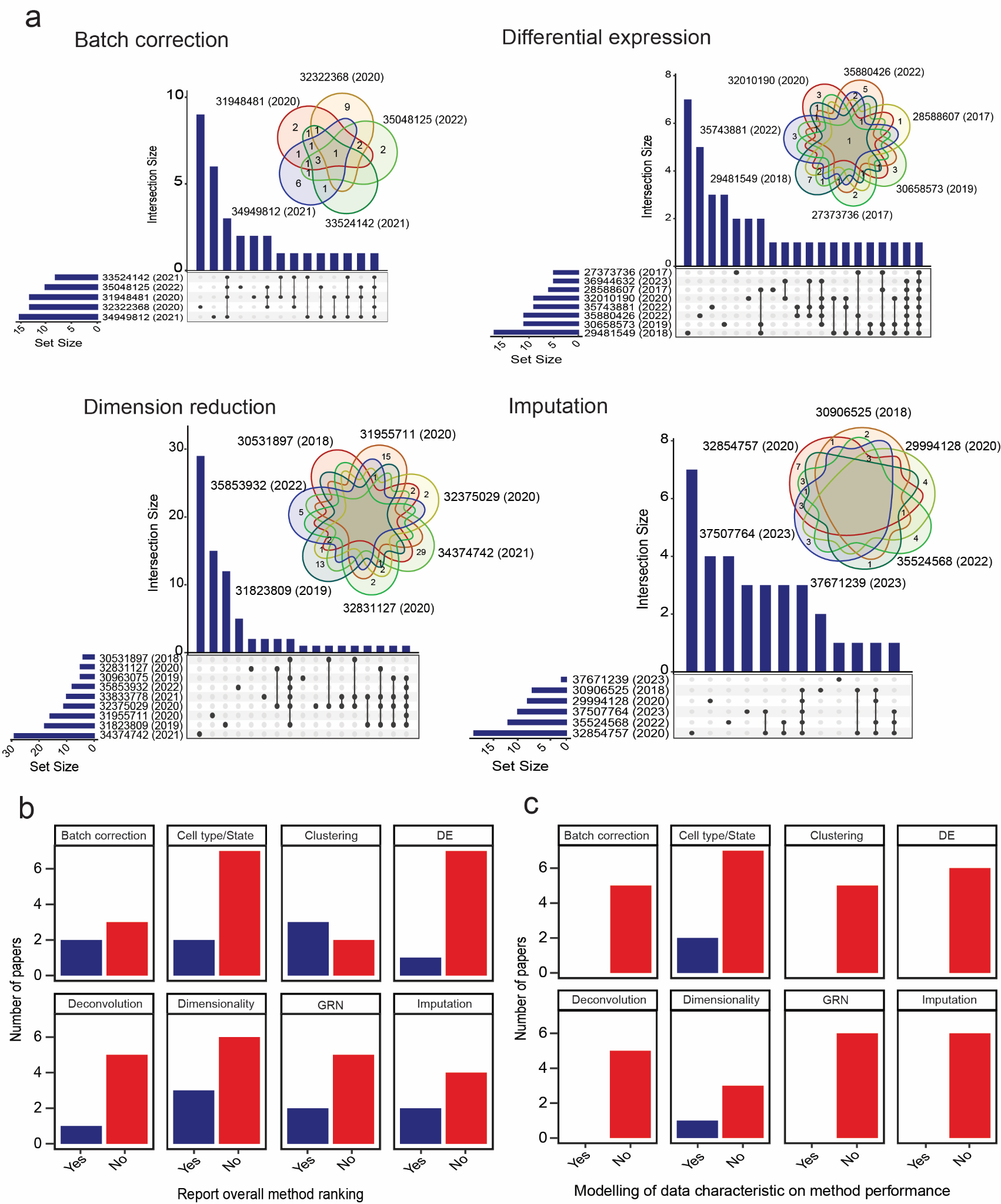


#### Supplementary Figure 6. Inspecting consistency across multiple benchmarking studies.

Topics with more than three benchmarking papers were assessed. a. Venn diagram and upset plot illustrate the common methods evaluated across multiple benchmarking papers within each category. Each paper is denoted by its PMID. There is considerable variability in the methods included, with only a few common methods across papers within the same topics, highlighting challenges in cross-study comparability. b. Number of papers that report the overall method ranking in each category. As shown by the plot, this practice is not common. c. Number of papers that analysed the impact of data characteristics on method performance in each category. While data has been reported as a key factor affecting method performance, most papers do not incorporate such modelling, suggesting potential of future examination. In b and c, “Cell type/State” refers to “Cell type/State identification”, “DE” refers to “Differential expression”, “Dimensionality” refers to “Dimension reduction”, “GRN” refers to “regulatory network inference”.


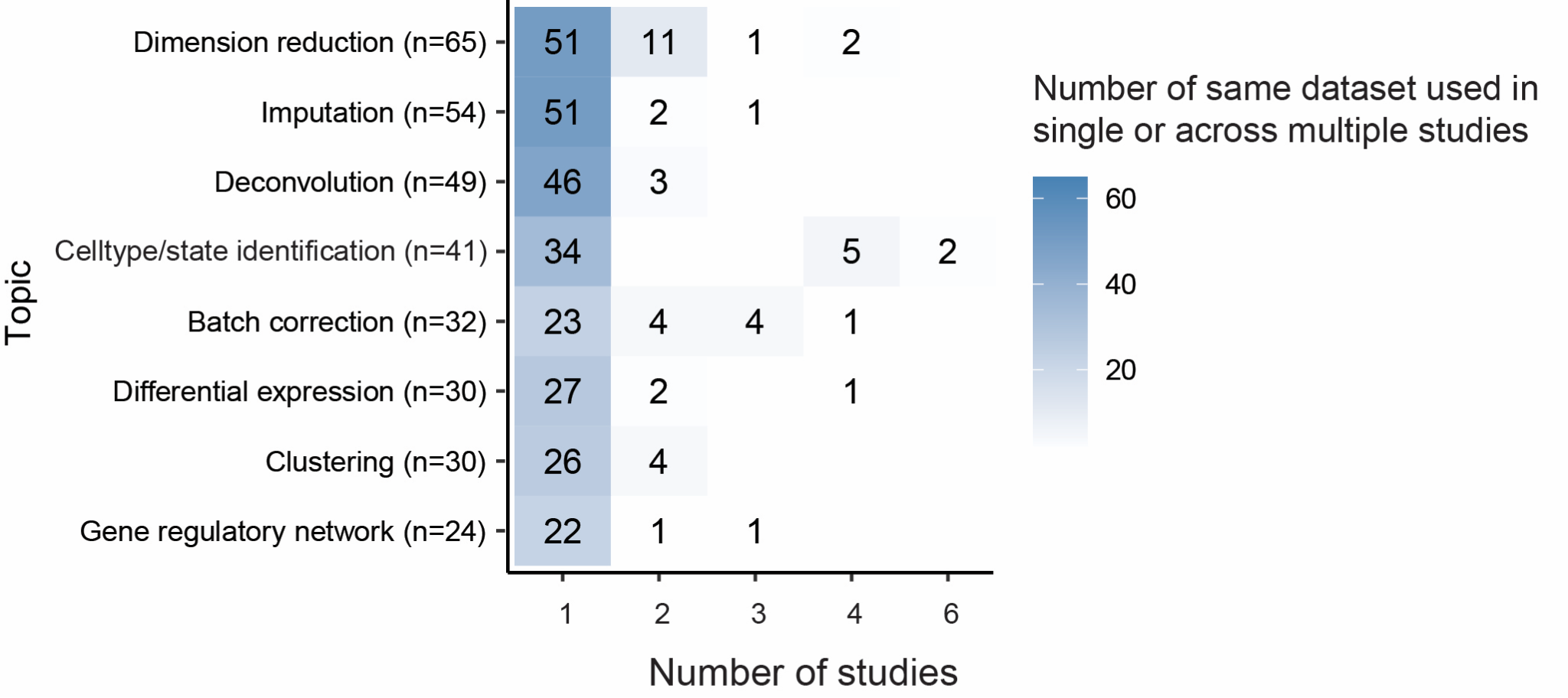


#### Supplementary Figure 7. Overlap of the datasets used by multiple benchmarking studies.

Topics with more than three benchmarking papers in the single-cell RNA-sequencing data type were assessed. The heatmap shows the number of times that each dataset was reused across studies. For example, across all dimension reduction papers, 65 unique datasets are used in total. Out of these 65 datasets, 51 datasets appeared in a single study, meaning the majority of datasets were not reused by other studies; 11 datasets appeared in two studies; one dataset appeared in three studies and two datasets appeared in four studies. Overall, across all topics, most datasets are used only once. This limited dataset reuse highlights the challenge in performing meta-analysis across studies on the same topic.


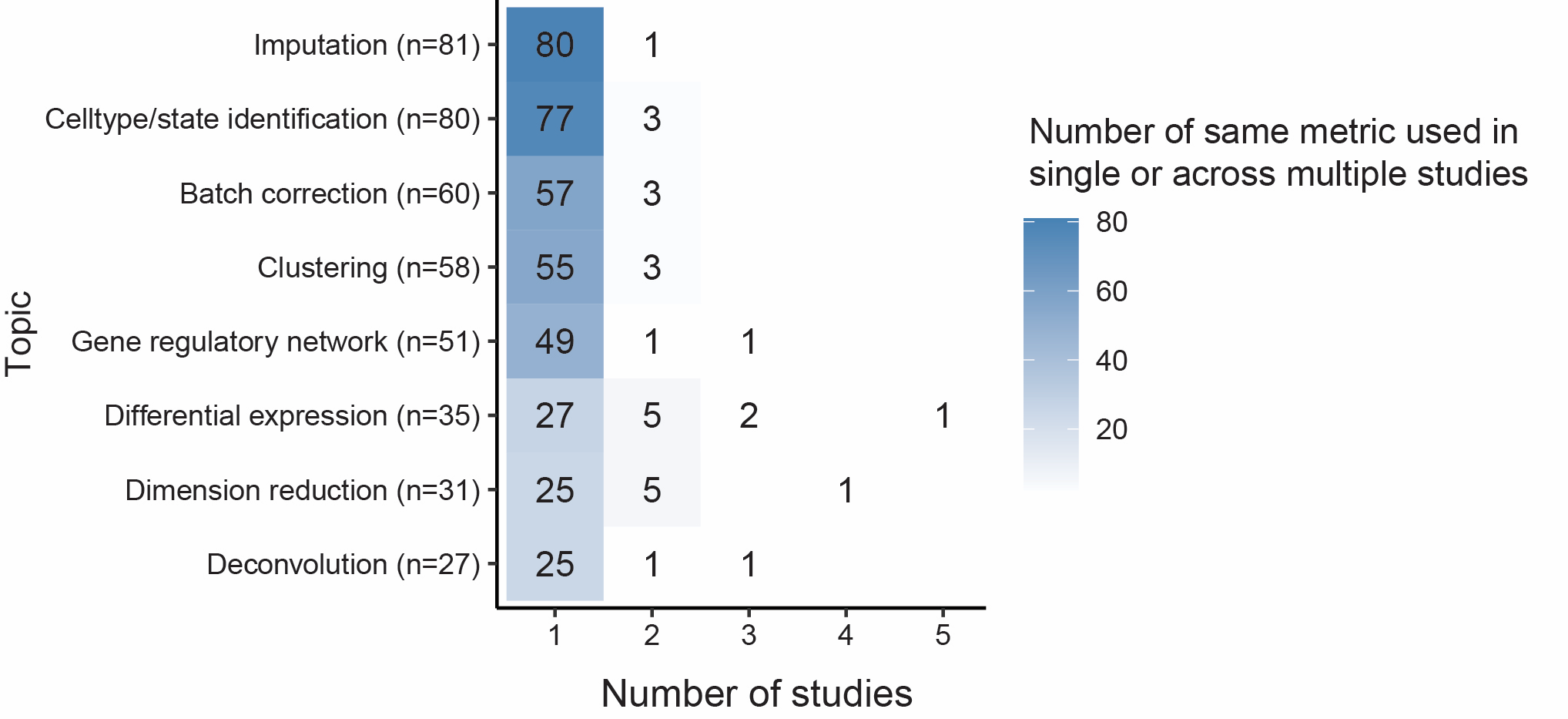


#### Supplementary Figure 8. Overlap of the statistical assessment metrics used by multiple benchmarking studies.

Topics with more than three benchmarking papers in the single-cell RNA-sequencing data type were assessed. The heatmap shows the number of times that each metric was reused across studies. For example, across all dimension reduction papers, 81 unique datasets are used in total. Out of these 81 metrics, 80 metrics appeared in a single study, meaning the majority of metrics were not reused by other studies and 1 dataset appeared in two studies. Overall, across all topics, most metrics are used only once. This limited metric reuse highlights the challenge in performing meta-analysis across studies on the same topic.

####
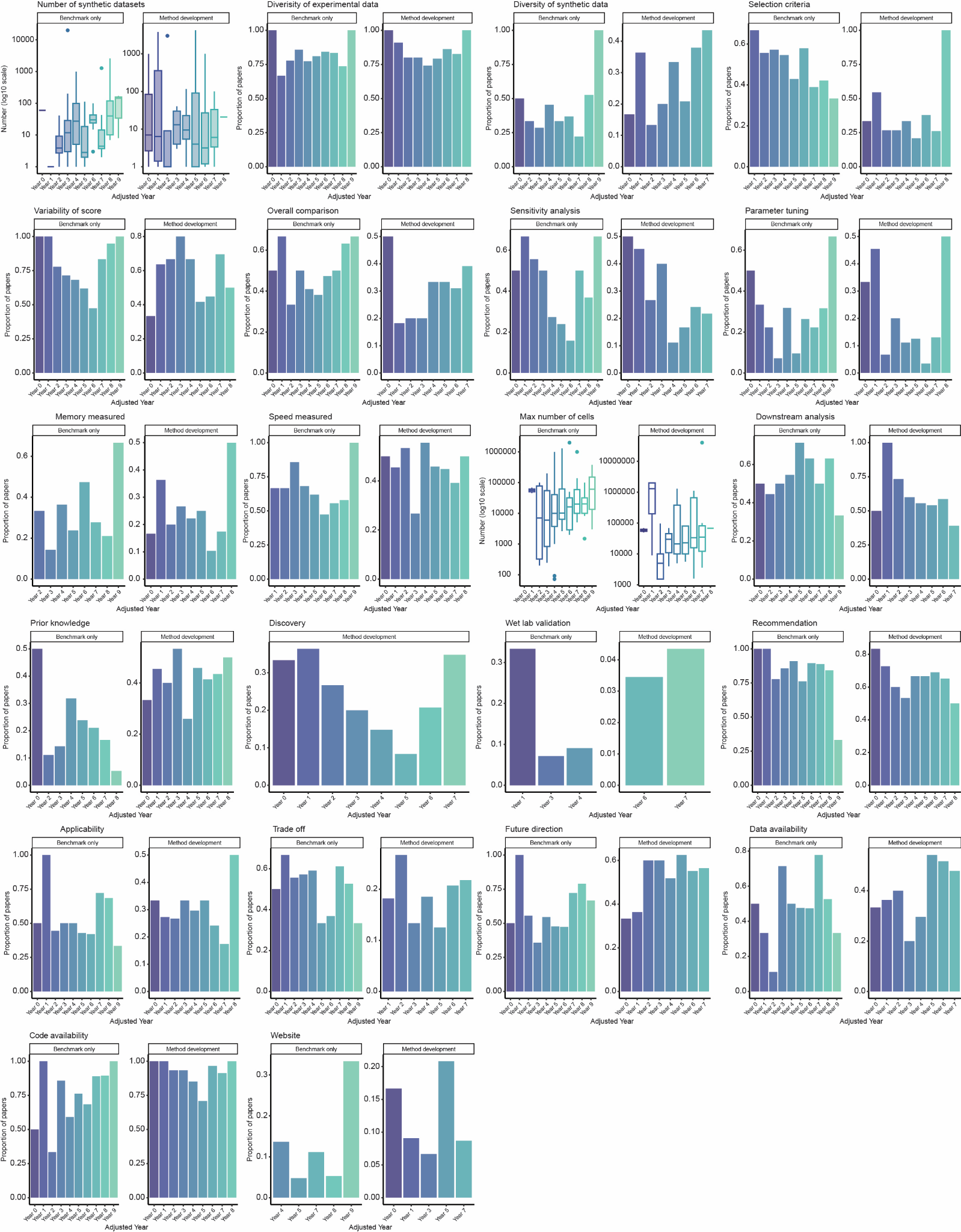
Supplementary Figure 9. Trends of variables over adjusted year.

This figure provides a visual overview of the evaluation criteria across the nine categories over the adjusted years. In BOP there is a clear increase in the number of number of synthetics data used, as well as an increase in the dataset size in scalability experiment as seen in the maximum number of cells used. For other variables, the trend across adjusted years varies.


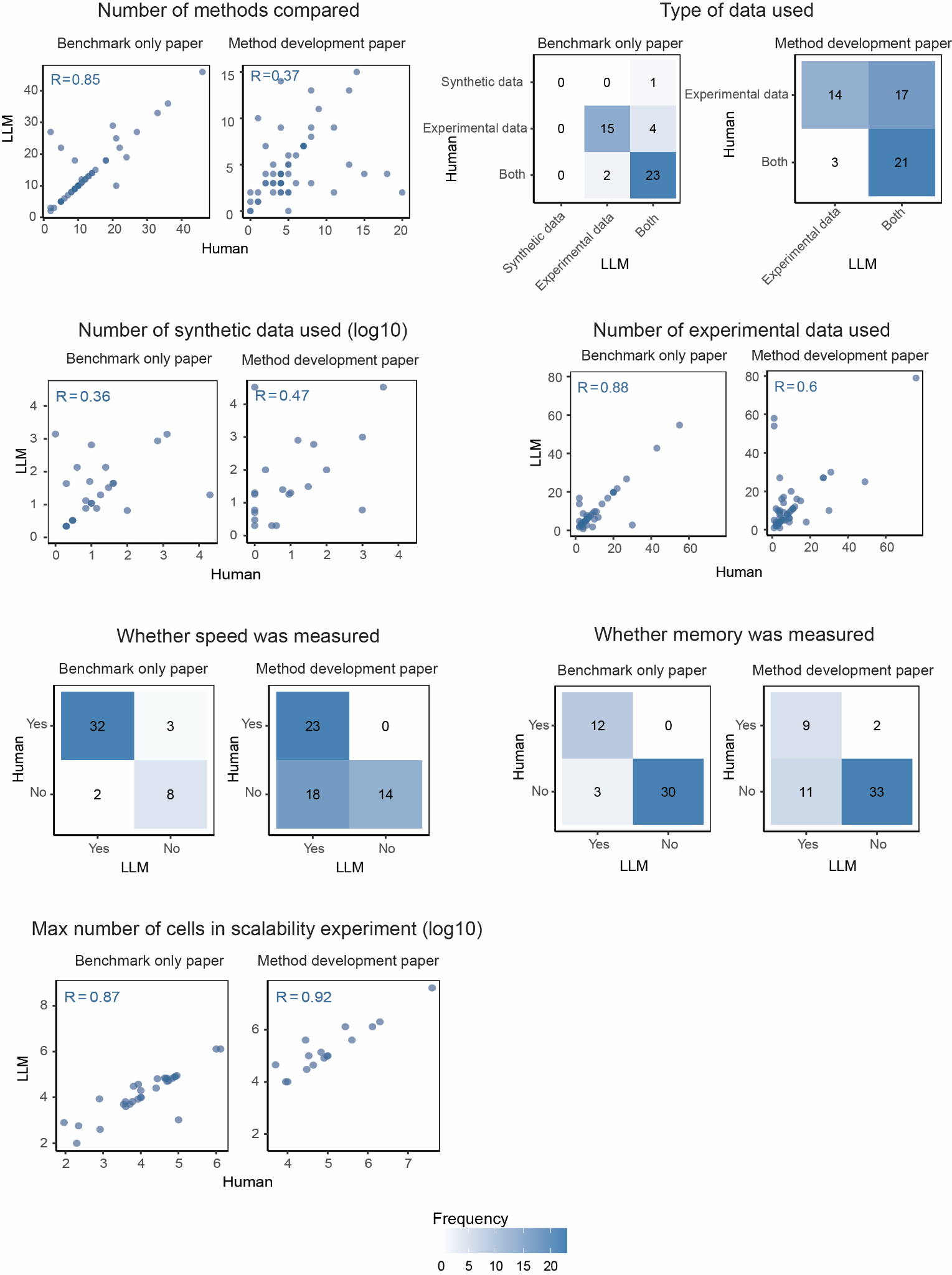


#### Supplementary Figure 10. Comparison of human response versus LLM response on selected quantitative variables.

Some variables were log10 transformed due to the broad range of values and for ease of visual interpretation. In those cases, Pearson’s correlation was calculated on the log10 transformed values. The maximum number of cells used in scalability experiments showed high correlation in both BOP (R = 0.87) and MDP (R = 0.92), likely because this information is typically explicitly reported in scalability plots, making extraction straightforward. We also observed a high correlation in the number of methods compared (R=0.85) and the number of experimental data used (R=0.88) for BOP. In contrast, only moderate correlations was observed for the number of methods (R=0.37) and number of experimental datasets (R=0.6) used in MDP. This is perhaps due to the less explicit ways that MDP present the comparative analysis compared with BOP. Similarly, the correlation of the number of synthetic data is only moderate for both BOP (R=0.36) and MDP (R=0.47). A possible reason is that synthetic data often involves various data resampling and parameter settings, and the exact dataset number is often not as explicitly reported as for experimental data.

### Supplementary Tables

#### Supplementary Table 1. Conversion of selected values into 0 to 1 for analytical purposes.

| Details of variables | Original response | After conversion |
| --- | --- | --- |
| All relevant variables involving “No”, “Yes” and “Not sure” | No | 0 |
|  | Yes | 1 |
|  | Not sure | 0.5 |
| Sensitivity | Default setting | 0 |
|  | Parameter tuning | 1 |
| Types of dataset | Experimental data | 0.5 |
|  | Synthetic data | 0.5 |
|  | Both | 1 |
| Number of experimental datasets Number of synthetic datasets  Number of methods compared  Max number of cells | Numeric value | Log10 normalisation, followed by scaling into [0, 1] |

##

## 
